# Alarmingly high recent human immunodeficiency virus infection burden among newly diagnosed individuals in Mongu District, Zambia

**DOI:** 10.4102/phcfm.v18i1.5202

**Published:** 2026-02-05

**Authors:** Masiliso A. Liamba, Nawa Mukumbata, Sepiso K. Masenga

**Affiliations:** 1 Ministry of Health, Mongu, Zambia; 2 Department of Epidemiology and Biostatistics, Faculty of Public Health, Levy Mwanawasa Medical University, Lusaka, Zambia; 3 Department of Physiological Sciences, School of Medicine, Mulungushi University, Livingstone, Zambia; 4 Department of Cardiovascular Science and Metabolic Diseases, Livingstone Center for Prevention and Translational Science, Livingstone, Zambia

**Keywords:** HIV, prevalence, recency, associated factors, Zambia

## Abstract

**Background:**

Human immunodeficiency virus (HIV) recency assays are essential for distinguishing recent infections (≤ 12 months) from long-term infections, enabling targeted interventions. Western province, Zambia, has the nation’s highest HIV prevalence (16%), yet recent infection data in Mongu District remain unknown.

**Aim:**

To determine the burden of recent HIV infection and associated factors in Mongu District, Western province, Zambia.

**Setting:**

The study was conducted in Mongu district, a provincial town in Western province in Zambia.

**Methods:**

This cross-sectional analytical study was conducted from 12 February 2024 to 30 May 2024 in 19 health facilities in Mongu District offering HIV testing services (HTS), using Asante recency test kits to identify recent infections. A total of 771 newly diagnosed individuals were included, and logistic regression analysis was employed to identify factors associated with recent infections, with adjusted odds ratio (AOR) and 95% confidence interval (CI) calculated, and significance determined at a *p*-value < 0.05.

**Results:**

Among 771 antiretroviral therapy (ART)-naïve individuals (median age 27 years; 71% female participants), 34% (*n* = 262) were identified as recent infections. Participants with medium (adjusted odds ratio [AOR] = 13.43, 95% confidence interval [CI]: 5.85–30.83) or low (AOR = 13.33, 95% CI: 5.67–31.33) HIV knowledge had higher odds compared to those with high knowledge (*p* < 0.001). Unsuppressed viral load (VL) (≥ 1000 copies/mL) showed the strongest association (AOR = 852.1, 95% CI: 521.1–1432; *p* < 0.001). Cluster of differentiation 4 (CD4) counts < 200 cells/mm^3^ had 1848-fold higher odds (AOR = 1848.2, 95% CI: 192.8–17714.1; *p* < 0.001). Clients who entered through the Prevention of Mother-to-Child Transmission had higher odds (adjusted odds ratio [AOR] = 2.67, 95% confidence interval [CI]: 1.11 – 6.11, *p* = 0.028) compared to the reference group.

**Conclusion:**

Mongu District exhibits alarmingly high recent HIV infections (34%), concentrated among youth, women, and individuals with suboptimal knowledge.

**Contribution:**

These findings underscore the urgent need for scaling recency testing, strengthening youth-focused prevention, and improving HIV literacy as critical public health priorities.

## Background

Human immunodeficiency virus (HIV) remains a critical global public health challenge. As of 2024, it’s estimated that worldwide 40.8 million people were living with HIV, 2.42 million of whom were children below 19 years old.^1^ Although advancements in antiretroviral therapy (ART) have enhanced life expectancy, the early detection of recent HIV infections, defined as those acquired within the past 12 months, is critical for reducing transmission and achieving epidemic control.^2,3^ The World Health Organization (WHO) recommends integrating recency testing into national HIV programmes to distinguish recent infections from long-term infections, enabling targeted prevention strategies. Zambia adopted recency testing in 2020, yet data on recent infections in high-prevalence regions such as Mongu District remain sparse.

Western province has an overall HIV prevalence of 16% Zambia Population-based HIV Impact Assessment (ZAMPHIA), exceeding the national average of 11.6%.^4^ Mongu, the provincial capital, is presumed to contribute substantially to this burden, yet no data exist on the prevalence of recent HIV infections within the district. Comparatively, HIV prevalence in other districts ranges from 17.2% in Choma and 15.0% in Livingstone to lower rates such as 4.8% in Kabompo and 4.3% in Lundazi.^4^

The lack of data on recent infections in Mongu District, a key indicator of ongoing transmission, limits policymakers’ and programme implementers’ ability to design effective, localised interventions. Understanding who is acquiring HIV now, and under what circumstances, is crucial for interrupting transmission networks and accelerating progress towards Zambia’s 95-95-95 targets.

This study seeks to address this gap by estimating the prevalence of recent HIV infections among newly diagnosed individuals in Mongu District, and identifying the sociodemographic, clinical and behavioural factors associated with recency. The findings will generate actionable evidence to strengthen prevention efforts in this high-prevalence setting.

## Research methods and design

### Study design

This was a facility-based, cross-sectional analytical study conducted between 12 February 2024 to 30 May 2024 in Mongu District, Western province of Zambia. The district was purposively selected because of its high HIV burden and limited data on recent infections. A total of 19 out of 42 public health facilities offering HIV testing services (HTS) were included in the study. These facilities were selected based on high client volume, geographical coverage across the district and capacity to support recency testing. Participants included individuals newly diagnosed with HIV who had not initiated ART.

### Study setting

Mongu District is a provincial town in Western province in Zambia. It is located approximately 600 km from Lusaka, the capital city of Zambia. The district itself is surrounded by the Barotse floodplain and the Zambezi River. The main economic activities are fishing and agriculture. The district has an estimated population of 212 000 by 2024. The district has a total of 42 public health facilities, comprising 1 General hospital, 1 district hospital, 3 mini hospitals, 20 health centres and 17 health posts, offering primary health care services. During the rainy season, people move from the remote floodplain to the upper land, which is the dry land. Despite these challenges, the district has a robust community health system.

### Study population and eligibility criteria

The study population comprised individuals aged 15 years and older, who were newly diagnosed with HIV during the study period. Eligible participants were those who had not yet initiated ART at the time of diagnosis, provided informed consent (and assent with parental or guardian consent for individuals aged below 18 years) and had no prior known HIV-positive result. Individuals unable to provide consent or assent, or those who had already started ART were excluded.

### Sample size

Using this formula for estimating a single proportion with 95% confidence, a conservative prevalence assumption of 50% (maximising required sample size), and a margin of error of ± 3.5%, the minimum sample size required was calculated as 784. The final sample of 771 provided a margin of error of ± 3.53%, which was deemed methodologically acceptable for prevalence estimation.

### Sampling method

A multistage cluster sampling approach was employed. Health facilities were first stratified by HIV testing volume (high, medium, low), and a proportionate number were randomly selected. Within each facility, consecutive sampling was applied to recruit eligible newly diagnosed individuals until the target sample size was reached.

### Study variables

The primary outcome variable was HIV recency status (recent vs. long-term). Human immunodeficiency virus recency test is a laboratory-based test that detects whether an HIV infection is recent (≤ 12 months) or not. This is carried out on newly HIV diagnosed clients not yet initiated on treatment. The incorporation of HIV recency testing in national HIV case reporting systems will help to assess how HIV is being transmitted, describe behaviours that are facilitating HIV transmission and optimise HIV-related data collection and information on risk factors. The outcome variable in this analysis was HIV recency, a categorical variable that differentiates between recent and long-term HIV infections based on testing criteria. The independent variables included socioeconomic status (income, education, and occupation) and demographic factors, including age and sex.

### Data collection and laboratory testing

Sociodemographic data were collected using structured questionnaires. Recency Testing Procedures: Recency testing was performed using the Asante HIV-1 Rapid Recency Assay, manufactured by Sedia Biosciences Corporation (Portland, Oregon, United States). The assay differentiates recent (≤ 12 months) from long-term infections by detecting the maturation of HIV-1 antibodies. The sensitivity and specificity of the Asanté HIV-1 rapid recency assay in diagnosing established HIV infection compared to the enzyme-linked immunosorbent assay (ELISA) were 98.4% (95% confidence interval [CI]: 96.7–99.3) and 99.6% (95% CI: 97.6–100.0), respectively.^5^

Blood samples were analysed using the Asante™ Recency Assay as part of the Recent Infection Testing Algorithm (RITA), which classifies infections as recent (< 12 months) or long-term by integrating Asante assay results, viral load (VL) (unsuppressed ≥ 1000 copies/mL), and clinical history. Collected blood specimens were analysed using a RITA. For HIV recency laboratory testing, blood collection was done using ethylenediaminetetraacetic acid (EDTA) collection (tubes with purple tops) by a qualified Biomedical Scientist with the help of the study nurse. For VL, results < 30 copies/mL were reported as Target Not Detected (TND).

### Data analysis

Data were analysed in Stata 14 (StataCorp LLC, College Station, Texas, United States). Descriptive statistics summarised participant characteristics. Chi-square or Fisher’s exact tests compared proportions, while logistic regression identified factors associated with recency. Adjusted odds ratios (AORs) with 95% CI were reported; statistical significance was set at *p* < 0.05.

### Ethical considerations

Ethical clearance was obtained from the ethical committee Excellence in Research Ethics and Science (ERES) Converge, and Institutional Review Board (IRB) Committee approved the study on 08 February 2024 (ethics reference number 2024-Jan-013). Mongu District Office gave permission to access the patients and collect samples. All participants provided written consent before participating in the study. All data collected and analysed were de-identified to ensure complete confidentiality, and no data were collected that could potentially identify any participants. Data security was maintained through password protection of all files and back-up on external media kept under lockable cabinets.

## Results

**FIGURE 1:**
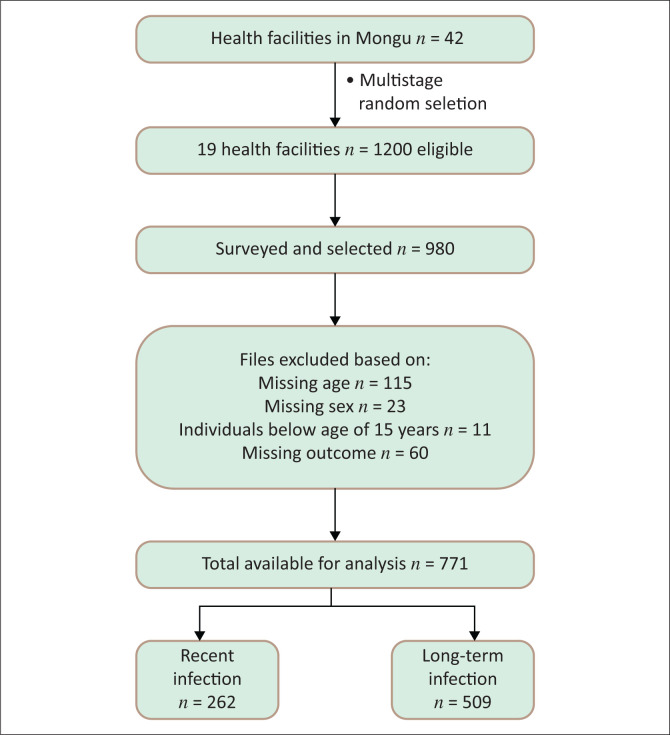
Multistage cluster sampling with consecutive recruitment (*n* = 771; recent = 262, long-term = 509).

The study enrolled 771 participants with a median age of 27 years (interquartile range [IQR]: 22–35) and a median cluster of differentiation 4 (CD4) count of 275 cells/mm^3^ (IQR: 232–364) (Figure 1 and Table 1). Female participants constituted 71% (*n* = 548) of the cohort. Recent HIV infections were identified in 34% (*n* = 262) of cases.

**TABLE 1:** The relationship between human immunodeficiency virus recency and social demographics of participants.

Variable	Category	Frequency				Long-term				Recent				*p*-value
		*n*	%	Median	IQR	*n*	%	Median	IQR	*n*	%	Median	IQR	
CD4	-	-	-	256	222, 310	-	-	296	232, 364	-	-	200	154, 335	**< 0.001**
Age (years)	-	-	-	27	22, 35	-	-	32	26, 40	-	-	23	19, 24	**< 0.001**
Age-category (years)	15–19	92	12	-	-	26	27	-	-	66	72	-	-	**< 0.001**
	20–24	222	29	-	-	85	38	-	-	137	62	-	-	
	25–49	418	54	-	-	359	86	-	-	59	15	-	-	
	50 & above	39	5	-	-	39	100	-	-	0	0	-	-	
Sex	Female	548	71	-	-	329	60	-	-	219	40	-	-	**< 0.001**
	Male	223	29	-	-	180	80	-	-	43	19	-	-	
Entry point	Index	201	26	-	-	148	74	-	-	53	26	-	-	**< 0.001**
	Mobile	15	2	-	-	10	67	-	-	5	33	-	-	
	Other	58	8	-	-	32	55	-	-	26	45	-	-	
	PITC	255	33	-	-	181	71	-	-	74	29	-	-	
	PMTCT	98	13	-	-	43	44	-	-	56	56	-	-	
	VCT	144	18	-	-	95	66	-	-	49	34	-	-	
Recency	Long-term	509	66	-	-	-	-	-	-	-	-	-	-	-
	Recent	262	34	-	-	-	-	-	-	-	-	-	-	
Education	Primary	75	10	-	-	62	83	-	-	13	17	-	-	**< 0.001**
	Secondary	242	31	-	-	118	49	-	-	124	51	-	-	
	Tertiary	454	59	-	-	329	72	-	-	125	26	-	-	
Employment	Employed	342	44	-	-	306	89	-	-	36	11	-	-	**< 0.001**
	Not Employed	429	56	-	-	203	47	-	-	226	53	-	-	
Marital status	Married	92	12	-	-	82	89	-	-	10	11	-	-	**< 0.001**
	Single	679	88	-	-	427	63	-	-	252	37	-	-	
HIV knowledge	Low	215	28	-	-	105	21	-	-	110	42	-	-	**< 0.001**
	Medium	134	17	-	-	43	8	-	-	91	35	-	-	
	High	422	55	-	-	3601	71	-	-	61	23	-	-	
	Suppressed	519	67	-	-	506	98	-	-	10	2	-	-	**< 0.001**
Viral load	Unsuppressed	252	33	-	-	3	2	-	-	252	100	-	-	-
CD4	< 200	129	17	-	-	1	1	-	-	128	99	-	-	**< 0.0001**
	> 200	642	83	-	-	508	79	-	-	134	21	-	-	

Note: PMTCT clinics identified the highest proportion of recent cases (56.1%), followed by other (45%). Data in bold signficant *p*-value < 0.05.

CD4, cluster of differentiation 4; IQR, interquartile range; PMTCT, Prevention of Mother-to-Child Transmission; VCT, voluntary counselling and testing; HIV, human immunodeficiency virus; PITC, Provider-Initiated Testing and Counselling.

### Sociodemographic and clinical associations

Recent infections were disproportionately higher among female participants (40.1% vs. 19.0% in male participants; *p* < 0.001) and younger individuals aged 15–24 years (73% vs. 14% in those aged 25–49 years; *p* < 0.001) (Table 1). Unemployment (53% vs. 11% employed; *p* < 0.001) and single marital status (37% vs. 11% married; *p* < 0.001) were strongly linked to recency. Participants with medium HIV knowledge had higher recency rates (67.9%) compared to those with high knowledge (14.4%; *p* < 0.001).

### Viral load and entry points

Prevention of Mother-to-Child Transmission (PMTCT) clinics identified the highest proportion of recent cases (56.1%), followed by voluntary counseling and testing (VCT) (34.3%).

### Logistic regression analysis

In the univariable analysis, older age was linked to lower odds of recent infection, with individuals aged 25–49 years significantly less likely to have a recent infection compared to those aged 15–19 years (OR = 0.06, 95% Cl: 0.03–0.10, *p* < 0.001) (Table 2). Male participants had lower odds of HIV recency compared to female participants (OR = 0.35, 95% Cl: 0.23–0.50, *p* < 0.001), while unemployment (OR = 9.61, 95% Cl: 6.48–14.27, *p* < 0.001) and being single (OR = 4.83, 95% Cl: 2.46–9.50, *p* < 0.001) were associated with significantly higher odds of recent infection. Participants diagnosed through Prevention of Mother-to-Child Transmission (PMTCT) had increased odds of HIV recency compared to those identified through index testing (OR = 3.60, 95% Cl: 2.15–5.93, *p* < 0.001). Education level also played a role, with secondary (OR = 5.17, 95% Cl: 2.70–9.88, *p* < 0.001) and tertiary education (OR = 1.88, 95% Cl: 1–3.53, *p* = 0.049) linked to higher odds of recent infection compared to primary education. Participants with low (OR = 6.19, 95% Cl: 4.23–9.07, *p* < 0.001) and medium (OR = 12.52, 95% CI: 7.96–19.69) HIV knowledge had increased odds of recency compared to those with high knowledge. Individuals who were single had 4.83 times higher odds of recent infection compared to those who were married (OR = 4.83, 95% CI: 2.46–9.50, *p* < 0.001). The strongest associations with recent HIV infection were observed for VL and CD4 count, although the extremely wide CIs indicate statistical uncertainty, likely because of the strong association and the sample size.

**TABLE 2:** Univariable and multivariable analysis of factors associated with human immunodeficiency virus recency.

Variable	Category	Unadjusted OR	*p*-value	95% CI	Adjusted OR	*p*-value	95% CI
CD4	Median (IQR)	0.99	**< 0.001**	0.98–98.00	1.000	0.882	0.99–1.00
Age (years)	**Median (IQR)**	**0.79**	**< 0.001**	**0.76–0.82**	**0.840**	**0.001**	**0.74–0.93**
Age-category (years)	15–19	15.44	**< 0.001**	9.08–26.26	0.750	0.720	0.14–3.83
	20–24	9.81	**< 0.001**	6.66–14.43	1.010	0.980	0.28–3.66
	25–49	**REF**	-	-	**REF**	-	-
	50 & above	1.00	-	-	1.000	-	-
Sex	Female	**REF**	-	-	**REF**	-	-
	Male	0.35	**< 0.001**	0.24–0.52	1.801	0.134	0.83–3.90
Entry Point	Index	**REF**	-	-	**REF**	-	-
	Mobile	1.39	0.559	0.46–4.27	0.340	0.318	0.04–2.80
	Other	2.26	0.008	1.23–4.15	0.730	0.579	0.24–2.17
	PITC	1.14	0.473	0.75–1.72	0.750	0.433	0.36–1.53
	PMTCT	3.60	**< 0.001**	2.15–5.93	2.670	0.028	1.11–6.44
	VCT	1.44	0.125	0.90–2.29	1.260	0.583	0.55–3.59
Education	Primary	0.55	0.065	0.22–1.04	0.330	0.096	0.09–1.21
	**Secondary**	**2.77**	**< 0.001**	**1.99–3.82**	**1.940**	**0.034**	**1.05–3.59**
	Tertiary	**REF**	-	-	**REF**	-	-
Employment	Employed	REF	-	-	REF	-	-
	Not employed	9.46	**< 0.001**	6.38–14.03	1.680	0.400	0.49–5.77
Marital Status	Married	REF	-	-	REF	-	-
	Single	4.83	**< 0.001**	2.46–9.50	2.090	0.341	0.45–9.61
HIV knowledge	**Low**	**6.19**	**< 0.001**	**4.23–9.07**	**13.330**	**< 0.001**	**5.67–31.33**
	**Medium**	**12.52**	**< 0.001**	**7.96–19.69**	**13.430**	**< 0.001**	**5.85–30.83**
	High	REF	-	-	REF	-	-
Viral load	Suppressed	REF	-	-	REF	-	-
	**Unsuppressed**	**178.10**	**< 0.001**	**104.00–720.00**	**852.1000**	**< 0.001**	**521.1–1432.00**
CD4	**< 200**	**485.10**	**< 0.001**	**67.20–3503.30**	**1848.200**	**< 0.001**	**192.8–17714.10**
	> 200	REF	-	-	REF	-	

Note: The variables in bold are statistically significant in both unadjusted and adjusted.

OR, odds ratio; CI, confidence interval; CD4, cluster of differentiation 4; IQR, interquartile range; PMTCT, prevention of mother-to-child transmission; VCT, voluntary counselling and testing; HIV, human immunodeficiency virus; PITC, Provider-Initiated Testing and Counselling; REF, reference.

However, the wide CI (including on multivariate analysis) suggests that while the effect is strong, there is some uncertainty in the precise estimate. On multivariable analysis CD4 count, age, education, PMTCT entry point knowledge about HIV and VL remained significantly associated with HIV recency, emphasising the critical role of demographic, socioeconomic and clinical factors in understanding HIV recency.

After adjusting for potential confounders, several variables remained significantly associated with recent HIV infection. Individuals with low and medium HIV knowledge had markedly higher odds of recent infection compared to those with high knowledge, with AORs of 13.33 (95% CI: 5.67–31.33, *p* = 0.005) and 13.43 (95% CI: 5.85–30.83), respectively. An unsuppressed VL was the strongest predictor, with individuals having an AOR of 852.1 (95% CI: 521.1–1432, *p* < 0.001) for recent infection. Those with secondary education had increased odds of recent infection (AOR = 1.94, 95% CI: 1.05–3.59, *p* = 0.034), and entry through PMTCT entry point was also a significant predictor (AOR = 2.67, 95% CI: 1.05–3.59, *p* = 0.028). Conversely, older age was protective (AOR = 0.08, 95% CI: 0.74–0.93, *p* < 0.001), while individuals with CD4 counts below 200 cells/mm^3^ had dramatically higher odds of recent infection (AOR = 1848.2, 95% CI: 192.8–177 714.1, *p* < 0.001).

## Discussion

A total of 771 participants were included in this study. The findings reveal a high incidence of recent HIV infections (34%) in Mongu District, signalling active transmission despite Zambia’s scale-up of ART. The rate exceeds estimates from Uganda (17.0%), Kenya (8.6%) and Malawi (19.7%), potentially reflecting regional disparities in testing coverage or epidemic dynamics.^6,7,8^ Notably, the predominance of recent infections among youth and women aligns with global trends, underscoring the vulnerability of these groups to transactional relationships and limited access to prevention services.^9^

Participants with low and medium levels of HIV knowledge exhibited higher odds of recent HIV infection than those with high HIV knowledge. This finding suggests that limited understanding of HIV transmission and prevention may contribute to increased vulnerability to new infections. It highlights the importance of not only providing accurate HIV-related information but also ensuring that it is accessible, contextually relevant and translated into preventive behaviours.^10^

Individuals with secondary education had significantly higher odds of recent HIV infection compared to those with tertiary education. This finding, while seemingly counterintuitive, is consistent with findings in Zambia^11,12,13^ and Malawi,^14^ where secondary education has been associated with elevated HIV risk. The increased odds among secondary-educated individuals may reflect a combination of limited but overestimated knowledge, increased sexual exposure and socioeconomic pressures that do not translate into true behavioural empowerment, as observed in other sub-Saharan African contexts.^15,16^

Furthermore, individuals with CD4 counts below 200 cells/mm^3^ had dramatically higher odds of recent infection. Consistent with established recency-incidence models, a CD4 count below 200 cells/mm^3^ at diagnosis is strongly indicative of recent HIV infection, as such low counts are unlikely in clients established on treatment. This aligns with findings from Brazilian recency-score models, where lower CD4 counts were weighted as strong predictors of recent infection. These models recognise that significant CD4 depletion often occurs shortly after seroconversion, particularly in cases of rapid disease progression. Therefore, markedly low CD4 counts at diagnosis may reflect acute or early-stage HIV.

### Strengths and limitations

The calculated sample size was 784, of which 771 participants were analysed. Eleven were excluded for being below the eligible age of 15 years, and two were excluded because of missing age data. Despite the large and diverse sample, the study had some limitations. The exclusion of participants because of age ineligibility and missing data may have introduced minor selection bias. However, cross-sectional data preclude causal inferences, and facility-based recruitment may underrepresent marginalised populations. Self-reported behaviours and knowledge levels could introduce bias. Silent transfers (undisclosed prior HIV+ status) may cause misclassification of long-term infections as recent. The extremely wide CIs observed for key predictors such as CD4 count and unsuppressed VL are partly attributable to the strong association and the sample size, which, while large, resulted in small cell counts for some outcomes (e.g. very few long-term infections had unsuppressed VL). While these strong associations align with biological plausibility (low CD4/viremia typifying recent infection), the imprecision in effect sizes limits clinical interpretability. We acknowledge this as a key methodological limitation.

### Clinical and public implications

The higher proportion of recent HIV infections observed in PMTCT compared to VCT may be attributed to differences in target populations and testing schedules. Pregnant and breastfeeding women are routinely tested every three months during pregnancy and throughout the breastfeeding period, which increases the chances of detecting HIV infection early, a finding supported by studies in Ethiopia.^17^ However, low male partner involvement in PMTCT programmes may contribute to the ongoing risk of seroconversion during pregnancy or breastfeeding, particularly within marital and cohabiting relationships. In contrast, individuals visiting VCT centres are typically tested only once and often do so when symptoms appear. This shows that gaps are still there in early detection of HIV outside PMTCT and highlights the need for active male involvement and enhanced other testing modalities such as community and index testing. To address this, more targeted interventions are needed to improve testing uptake in VCT centres, including community-based mobile testing and social network strategies to reach individuals who do not routinely access facility-based services. Findings advocate for targeted interventions, including youth-focused education campaigns, expanded pre-exposure prophylaxis (PrEP), and integration of recency testing into routine surveillance.

## Conclusion

Mongu District exhibits an urgent epidemic of recent HIV infections (34%), concentrated among youth and individuals with limited HIV knowledge. This translates to a critical need for targeted HIV programmes such as PrEP strategies, condom promotion, and more youth support programmes to reduce the ongoing transmission, such as school-based comprehensive sexuality education and communication with children on safer sex, Nevertheless, these findings unequivocally support scaling three evidence-based actions: (1) integration of recency testing into routine surveillance, (2) targeted prevention for youth/women through PMTCT/VCT platforms, and (3) community-led HIV literacy programmes.
